# The time to care is now: a multidisciplinary call to action for addressing paediatric burn injuries globally

**DOI:** 10.1093/inthealth/ihaf035

**Published:** 2025-04-11

**Authors:** Kajsa Vlasic, Theresia Mwakyembe, Irma Fleming, Giavonni Lewis, Sudha Jayaraman, Catherine A Staton, Melissa H Watt, Blandina T Mmbaga, Elizabeth M Keating

**Affiliations:** Division of Pediatric Emergency Medicine, Department of Pediatrics, University of Utah, Williams Building, 295 Chipeta Way, Salt Lake City, Utah, USA 84108; Kilimanjaro Christian Medical Centre, Box 3010, Moshi, Tanzania; Kilimanjaro Christian Medical University College, Box 2240, Moshi, Tanzania; Department of Surgery, University of Utah, 30 Mario Capecchi Drive, Salt Lake City, Utah, USA 84112; Department of Surgery, University of Utah, 30 Mario Capecchi Drive, Salt Lake City, Utah, USA 84112; Center for Global Surgery, Department of Surgery, University of Utah, 30 Mario Capecchi Drive, Salt Lake City, Utah, USA 84112; Department of Emergency Medicine, Duke University Medical Center, 2301 Erwin Road, Durham, North Carolina, USA 27710; Global Emergency Medicine Innovation and Implementation Research Center, Duke University, 2301 Erwin Road, Durham, North Carolina, USA 27710; Duke Global Health Institute, Duke University, Box 90519, Durham, North Carolina, USA 27708; Department of Population Health Sciences, University of Utah, Williams Building, 295 Chipeta Way, Salt Lake City, Utah, USA 84108; Kilimanjaro Christian Medical Centre, Box 3010, Moshi, Tanzania; Kilimanjaro Christian Medical University College, Box 2240, Moshi, Tanzania; Global Emergency Medicine Innovation and Implementation Research Center, Duke University, 2301 Erwin Road, Durham, North Carolina, USA 27710; Kilimanjaro Clinical Research Institute, Box 2236, Moshi, Tanzania; Division of Pediatric Emergency Medicine, Department of Pediatrics, University of Utah, Williams Building, 295 Chipeta Way, Salt Lake City, Utah, USA 84108

Childhood burn injuries are life-changing, leaving lasting physical and emotional scars that profoundly impact physical functioning, self-esteem, social interactions and future opportunities. Whether the injury is caused by an act of war or a cooking incident at home, the consequences are equally devastating for children and their families. Addressing the multiple challenges of paediatric burn injuries requires multidisciplinary coordination to ensure timely access to evidence-based care and comprehensive support for physical recovery, emotional healing and reintegration into daily life. In response to data from a paediatric injury registry implemented in northern Tanzania showing a large burden of in-hospital mortality for paediatric burn injury patients at a zonal referral hospital,^1^ we formed a collaborative multidisciplinary partnership of international experts to address this challenge. Our group has identified multiple barriers to prompt and high-quality burn care that require coordinated action to improve outcomes for paediatric patients.

As clinicians and public health researchers, our team observes first-hand the gravity of childhood burns and the impact of delayed or suboptimal care for these patients. When we care for a toddler with a scald burn injury in the critical care unit of a zonal referral hospital on the slopes of Mount Kilimanjaro in Tanzania, caregivers frequently recount details of a multiday transport with limited pre-hospital care and no formal ambulance system to assist. When we resuscitate an adolescent with traumatic burns from a motor vehicle crash in a resource-intensive level-one trauma emergency department in the mountains of Utah, we see the layers of transport our patient endured while crossing state borders to access care at the only regional paediatric subspecialty hospital. The complexities surrounding the provision of timely and effective treatment for burn patients are exacerbated in resource-poor areas of all countries by a multitude of factors along the continuum of care. The limitations range from a lack of prevention initiatives, to geographical restrictions and a lack of formal emergency transport systems impacting early burn trauma resuscitation, to limited location-specific and evidence-based in-hospital clinical care guidelines and the absence of formal rehabilitation services for those who survive their injuries. Strong multidisciplinary burn care involves intentional collaboration along the care continuum among clinical disciplines and in this case includes paediatricians, emergency physicians, burn surgeons, public health specialists with an understanding of health systems, mental health and social work support for patients and their families and rehabilitation experts.

Over recent decades, there has been a notable epidemiologic shift in global childhood deaths, with a relative increase in mortality from injuries and non-communicable diseases and a decrease from infectious diseases and malnutrition.^2^ The study of global paediatric injuries is increasing and a large burden of paediatric burn injuries has been identified.^3^ While burn injuries impact individuals of any age and in all corners of the world, burn injuries disproportionately impact children in low- and middle-income countries (LMICs), with the majority occurring in sub-Saharan Africa and Southeast Asia. Children who experience burn injuries in LMICs currently are seven times more likely to die than those injured in high-income countries (HICs).^4^ For those children who survive their burn injuries, there are other obstacles that impact quality of life and productive life—especially when access to subspecialist care is limited and difficult to reach.

Despite the growing evidence highlighting the increasing burden of paediatric burn injuries in resource-limited areas, notable knowledge gaps persist, including a shortage of high-quality studies to guide burn care in LMIC settings and skewed representation of research from certain geographic areas.^3^ Current literature shows that children who suffer all types of injuries in resource-limited settings often experience significant delays in reaching needed subspecialty care that likely contribute to worse outcomes and death.^5^ For those with burn injuries specifically, lack of specialized care and fragmented services across healthcare facilities contribute to poor outcomes. Children are especially at risk of emotional and psychological trauma associated with their burn injuries and treatment. As a global community of paediatric medical providers, it is imperative that we build a multidisciplinary community of experts focused on collaboration to improve paediatric burn injury care worldwide.

Given the global burden of paediatric burn injuries and the multifaceted challenges these children face, as a team of health experts focused on paediatric burn injuries we urgently call on the global health community to embrace these five critical actions moving forward.

## Develop evidence-based paediatric burn care guidelines specific to LMIC settings

The study of paediatric sepsis management in resource-limited settings in Africa was a landmark moment in recognizing that fluid bolus guidelines effective in HIC settings do not translate to African LMICs.^6^ Similarly, therapeutic and interventional studies are needed to inform evidence-based guidelines for resuscitation and management of paediatric burn injury patients in LMICs to optimize outcomes in these settings.

## Enable timely triage and organized referral for paediatric burn injury patients

Delays in care are well studied in many contexts and our understanding of their impact in LMIC paediatric injury populations is growing.^5^ Many individuals who live in resource-limited areas are impacted by a multitude of barriers in reaching definitive medical care. In hierarchical healthcare systems, families take their child to a local health post first, where the providers and setting may lack the resources to appropriately manage burns. Prompt resuscitation for burn injury patients leads to improved outcomes in HIC settings. Development and implementation of continuous education for paediatric burn injury care—including pre-hospital resuscitation, triage systems and proactive referrals for patients requiring higher levels of care—as well as prevention and community outreach programs will greatly impact outcomes in this population.^7,8^

## Perform local needs assessments to develop context-specific paediatric burn prevention and intervention efforts

Healthcare systems vary by country, and each comes with its own intricate details regarding referral systems, emergency transport services, insurance coverage and subspecialty care, including access to paediatric-specific and burn-specific treatment. Understanding the nuances of local healthcare systems and the needs of local patients is vital to develop contextually appropriate burn care prevention efforts and interventions. Needs assessments must include the voices of patients and their families, local healthcare workers and the experiences of pre-hospital, in-hospital and post-discharge clinical care.

## Ensure subspecialty and multidisciplinary care for complex burn injury patients

Paediatric care outcomes improve with subspecialty support, and burn injuries are no exception. Paediatric subspecialty care is limited in much of the world and many paediatric patients are treated by diligent healthcare workers who do not have specific expertise caring for infants, children and young adults. While formal fellowship education programs develop in LMICs—including paediatric emergency medicine, paediatric surgery and paediatric critical care medicine—there is a paramount need to work as multidisciplinary teams to support this complex injury population during their interface with current healthcare systems in low-resource areas. Increasing technical training for surgical staff focused on burns and reconstruction is also critical to ensure appropriate clinical care when these patients arrive at subspecialty facilities. The development of multidisciplinary teams needs to extend beyond expert groups of clinicians and include researchers, mental health specialists, public health professionals, public policy experts and funders to increase local capacity building.

## Renew our dedication to high-quality research

Most literature on paediatric burn injuries in LMIC settings is descriptive in nature and few articles exist addressing therapeutic interventions or long-term follow-up in this patient population.^3^ In addition to developing the evidence that drives clinical practice guidelines, as previously described, high-quality burn-specific research with local researchers and clinicians is necessary to improve our understanding of morbidity and mortality in this population. Taking an implementation science–focused approach will allow for improved understanding of the application of newly acquired evidence in low-resource settings. Understanding context-specific barriers through a variety of analysis modalities and then creating interventions, followed by adapting those interventions, requires unique strategies in different health system settings. Continuous re-evaluation of both the implementation process and the measure of clinical outcomes is vital to creating effective change in areas with limited data and limited resources.

The need for change is clear. It is time to break down silos, unite across disciplines and commit to advancing paediatric burn care across the globe.

## Data Availability

No new data were generated or analysed in support of this article.
